# The global depth range of marine fishes and their genetic coverage for environmental DNA metabarcoding

**DOI:** 10.1002/ece3.9672

**Published:** 2023-01-18

**Authors:** Agnès Duhamet, Camille Albouy, Virginie Marques, Stephanie Manel, David Mouillot

**Affiliations:** ^1^ MARBEC Univ Montpellier, CNRS, IRD, Ifremer Montpellier France; ^2^ CEFE Univ Montpellier, CNRS, EPHE‐PSL University, IRD Montpellier France; ^3^ Ecosystem and Landscape Evolution, Institute of Terrestrial Ecosystems, Department of Environmental Systems Science ETH Zürich Zürich Switzerland; ^4^ Unit of Land Change Science Swiss Federal Research Institute WSL Birmensdorf Switzerland; ^5^ Institut Universitaire de France Paris France

**Keywords:** deep‐sea, depth range, environmental DNA, genetic reference database, marine fishes, metabarcoding markers

## Abstract

The bathymetric and geographical distribution of marine species represent a key information in biodiversity conservation. Yet, deep‐sea ecosystems are among the least explored on Earth and are increasingly impacted by human activities. Environmental DNA (eDNA) metabarcoding has emerged as a promising method to study fish biodiversity but applications to the deep‐sea are still scarce. A major limitation in the application of eDNA metabarcoding is the incompleteness of species sequences available in public genetic databases which reduces the extent of detected species. This incompleteness by depth is still unknown. Here, we built the global bathymetric and geographical distribution of 10,826 actinopterygian and 960 chondrichthyan fish species. We assessed their genetic coverage by depth and by ocean for three main metabarcoding markers used in the literature: teleo and MiFish‐U/E. We also estimated the number of primer mismatches per species amplified by in silico polymerase chain reaction which influence the probability of species detection. Actinopterygians show a stronger decrease in species richness with depth than Chondrichthyans. These richness gradients are accompanied by a continuous species turnover between depths. Fish species coverage with the MiFish‐U/E markers is higher than with teleo while threatened species are more sequenced than the others. “Deep‐endemic” species, those not ascending to the shallow depth layer, are less sequenced than not threatened species. The number of primer mismatches is not higher for deep‐sea species than for shallower ones. eDNA metabarcoding is promising for species detection in the deep‐sea to better account for the 3‐dimensional structure of the ocean in marine biodiversity monitoring and conservation. However, we argue that sequencing efforts on “deep‐endemic” species are needed.

## Graphical abstract

Deep‐sea ecosystems are among the least explored on Earth due to their inaccessibility but are increasingly impacted by human activities. Environmental DNA (eDNA) metabarcoding has emerged as a promising method to study deep‐sea biodiversity but a major limitation in the application of eDNA metabarcoding is the incompleteness of species sequences available in public genetic databases which reduces the number of detected species. Here we have assessed the bathymetric distribution of 10,826 actinopterygian and 960 chondrichthyan species and the availability of sequences in public genetic databases along the depth gradient for the three principally used metabarcoding markers for fishes: teleo, MiFish‐U and MiFish‐E.

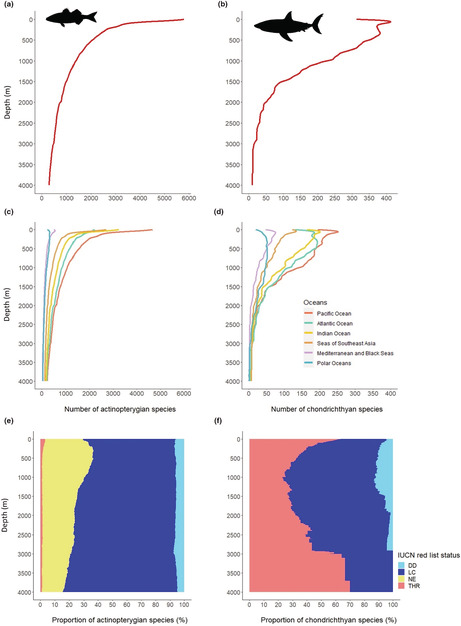

## 1 INTRODUCTION

The deep‐sea (>200 m depth) represents the largest biome on Earth (>65% of the Earth's surface, and >95% of the world's ocean volume) but is also the least explored with many undescribed species (Danovaro et al., 2020). Yet, despite the inherent difficulty of accessing this environment, the deep‐sea is increasingly threatened by multiple direct and indirect anthropogenic threats (Danovaro et al., 2020; Ramirez‐Llodra et al., 2011) such as fisheries (Clark et al., 2016), climate change (Levin & Le Bris, 2015), pollution (Jamieson et al., 2017), and hydrocarbon extraction or mining (Gollner et al., 2017; Jones et al., 2017). For example, there is a growing interest in harvesting the mineral deep‐sea resources to sustain “blue growth” and economic development (Wedding et al., 2015). However, these ecosystems are particularly sensitive to anthropogenic pressures (Niner et al., 2018). Indeed, the deep‐sea environment is considered as being largely pristine, slow to recover and hosting a high number of rare and threatened species (Niner et al., 2018; Sigwart et al., 2019). Anthropogenic pressures jeopardize biodiversity and ecosystem services provided by the deep‐sea such as the regulation of greenhouse gases, sea surface and atmospheric temperature or pollutants (Armstrong et al., 2012). In this context, the diversity of deep benthic and pelagic biota may experience a silent erosion with unknown consequences (Costello et al., 2012; Danovaro et al., 2014; Webb et al., 2010).

Deep‐sea ecosystem knowledge has been revolutionized by the discovery of high productivity habitats, such as seamounts (Clark et al., 2010) or chemosynthetic environments (Jannasch, 1985). Recent investigations have revealed that deep‐sea ecosystems are richer in species and more structured than previously thought owing to complex evolutionary history with multiple points of origin and radiations that may have been influenced by fluctuation of oxygen availability and temperature but also local patterns of oceanic circulation and habitat complexity (Danovaro et al., 2014; McClain & Hardy, 2010; Miller et al., 2022). Species composition varies widely along the water column and some species only occur at particular depths (Lesser et al., 2019; Macdonald et al., 2016; Stefanoudis, Gress, et al., 2019; Stefanoudis, Rivers, et al., 2019). For example, Lined hogfish (*Bodianus leucosticticus*) is a typical mesophotic fish living between 50‐ and 75‐m depth (Rocha et al., 2018). This information is key in conservation, particularly for the design and monitoring of Marine Protected Areas (MPAs) that should cover most species habitats and environments (Hanson et al., 2020) beyond the basic target of spatial coverage (10% in 2020 and 30% in 2030 for the marine realm) to safeguard or rebuild biodiversity (Duarte et al., 2020). So, marine conservation planning urgently needs to take into account the 3‐dimensional nature of the oceans and deep‐sea ecosystems (Levin et al., 2017). However, even if we have accumulated a large amount of knowledge on deep‐sea ecosystems in the last 130 years (McClain & Hardy, 2010; Paulus, 2021), we still misunderstand how their biodiversity is distributed due to its inaccessibility and large water volume (Everett & Park, 2018; Ramirez‐Llodra et al., 2011). Indeed, visual surveys with cameras are limited while fisheries data are often unreliable, biased, opportunistic, and sometimes forbidden in the deep‐sea (Rourke et al., 2022; Wormuth & Roper, 1983). Rare, elusive and cryptobenthic species are less detected than the others with these methods (Boussarie et al., 2018; Bozec et al., 2011). Furthermore, the use of Remotely Operated Vessels and Autonomous Underwater Vehicles in deep‐sea research remains too expensive and limited in space and time (Canals et al., 2021). Thus, new methods are urgently needed to fill this knowledge gap and monitor these deep‐sea and mesophotic ecosystems facing increasing disturbances (Paulus, 2021).

Environmental DNA metabarcoding has recently emerged as a promising non‐invasive approach to survey many marine taxa, complementing and often outperforming traditional inventory methods for detecting a wide breadth of species or Molecular Operational Taxonomic Units (MOTUs; e.g., Boussarie et al., 2018; Miya, 2022; Polanco Fernández, Marques, et al., 2021). Traditional methods like fishing nets and visual surveys are relevant to analyze fish size, maturity stage, abundance and behavior (McLean et al., 2018), while eDNA metabarcoding is complementary to these methods by improving species detection (Paulus, 2021; Ruppert et al., 2019). Environmental DNA (eDNA) is the DNA released by organisms in their environment through their mucus, shed skins or feces. eDNA is retrieved by water filtering and then analyzed using metabarcoding. For that, eDNA is amplified by Polymerase Chain Reaction (PCR) using markers designed to target different taxonomic groups (Ficetola et al., 2008; Miya, 2022). The amplified DNA fragments are sequenced and then assigned to species or taxa using available genetic databases (e.g., European Nucleotide Archive (ENA); Taberlet et al., 2012). eDNA metabarcoding has high potential to study and reveal deep‐sea biodiversity since it overcomes most difficulties inherent to classical methods (Brandt et al., 2021; Govindarajan et al., 2021; McClenaghan et al., 2020). For example, Visser et al. (2021) described deep‐sea cephalopod communities along the water column (between 50 and 1600 m depth) around Terceira Island using eDNA metabarcoding. Thomsen et al. (2016) demonstrated that eDNA metabarcoding can efficiently detect marine fishes in deep oceanic habitats. Moreover, Canals et al. (2021) used environmental DNA to assess fish species‐specific vertical distributions and movements through the water column such as the diel migratory behavior of some mesopelagic species. However, the major limitation to the large‐scale application of eDNA inventories is the incompleteness of species sequences available in public genetic databases which reduces the breadth of detected taxa (Deiner et al., 2017; Marques et al., 2021; Mathon et al., 2022; Ruppert et al., 2019). These knowledge gaps are particularly striking in megadiverse regions such as the neotropics where even well‐documented taxa such as marine fishes are poorly documented in genetic reference databases (Juhel et al., 2020). This is a major concern since fishes are among the most threatened vertebrates on Earth, particularly Chondrichthyes (Dulvy et al., 2021). Another limitation is the affinity of primers to the target sequences. Some species are well amplified by PCR with the selected metabarcoding markers while others are unlikely to be amplified due to mismatches between primers and target sequences reducing their detection probability (Elbrecht et al., 2018).

Thus, while the potential of deep‐sea eDNA metabarcoding to reveal new biodiversity patterns and refugia remains unachieved, it has the potential to drive new management actions or MPA establishments to reach conservation targets (Maxwell et al., 2020). Moreover, to our knowledge, neither the distribution of fish species richness (Actinopterygii and Chondrichthyes) nor their turnover according to depth (from shallow to bathypelagic layers) has ever been assessed at global scale. Such differences can be estimated through the number of species occupying a given depth (α‐diversity) but also by the turnover in species composition between depths (β‐diversity; Mittelbach & McGill, 2019). Here we took advantage of online resources such as FishBase (Froese & Pauly, 2022) and Ocean Biogeographic Information and System (OBIS, 2018) to build a global database of fish species depth range. Then, we assessed species genetic coverage by depth and oceanic basin for the three principal 12S mitochondrial primer pairs used in the literature: teleo (Valentini et al., 2016), MiFish‐U and MiFish‐E (Miya et al., 2015) which perform well to monitor fish biodiversity (Polanco Fernández, Richards, et al., 2021). The region targeted by the teleo primer pair is around 60 bp in length and is located at the end of the 12S mtDNA (Valentini et al., 2016). The region targeted by MiFish‐U primer pair is around 170 bp located at the beginning of the 12S mtDNA and is designed for actinopterygian sequence amplification (Miya et al., 2015). MiFish‐E primer pair targets the same region as MiFish‐U but is designed for chondrichthyan sequence amplification (Miya et al., 2015). We completed this study by analyzing primer affinity through the number of mismatches between each target sequence and the forward and reverse primers of each metabarcoding marker.

## 2 METHODS

### 2.1 Geographic distribution, depth range and IUCN status of marine fishes

Albouy et al. (2019) extracted the occurrence data (spanning the period 1826–2013) of 12,865 marine actinopterygian species from the OBIS database (https://obis.org) to construct their distribution map. These spatial data were structured on a 1° global grid. For Chondrichthyes, we used the Spatial Data & Mapping Resources of the IUCN Red List (downloaded in September 2021) containing polygons for 1127 marine Chondrichthyes species. We overlapped these polygons with a 1° resolution spatial grid, allowing us to determine species presence or absence in each 1° cell. We checked and updated all fish names using the WoRMS website (https://www.marinespecies.org) as the unique reference.

We retrieved the depth range of these marine fish species from FishBase (https://www.fishbase.se) in March 2022. Depth range was fully documented on FishBase for 9083 of these actinopterygian and 870 of these chondrichthyan species. To complete the minimum or maximum depth for the remaining fish species, we collected their occurrences from the OBIS database and intersected these occurrences with the bathymetric grid (etopo 1; NOAA, 2009) to determine the 1% and 99% quantiles of the bathymetric distribution for each species with at least 20 occurrences. We set these thresholds to avoid extreme, and sometimes unrealistic, values which can bias the estimation of species depth ranges.

Then, we applied different strategies to implement the missing depth range values. For demersal species, we considered 1% and 99% quantiles as the maximum and minimum depth, respectively. For bathydemersal species, we added the condition that the minimum depth had to be equal or deeper than 200 m. For reef‐associated species, we added the condition that the maximum depth had to be equal or shallower than 200 m. For bathypelagic species, the minimum depth had to be equal or deeper than 1000 m. For pelagic‐oceanic and benthopelagic species, we considered that the depth range is between the surface (0 m) and the 1% quantile. For pelagic‐neritic species, we added the condition that the maximum depth range had to be equal or shallower than 200 m. By applying these rules, we completed the bathymetric information for 1743 actinopterygian and 90 chondrichthyan species.

We downloaded the species IUCN red list status in September 2021 using the R package “rredlist” (Chamberlain, 2018) to assess the bathymetric distribution of threatened species and their genetic sequence coverage for eDNA metabarcoding according to depth. We grouped together Near Threatened, Vulnerable, Endangered and Critically endangered in one category called Threatened/Near Threatened (THR). Each species was assigned to one of these four IUCN Red list categories: Data Deficient (DD; for which data are insufficient to evaluate their conservation status), Not evaluated (NE), Least Concern (LC) and THR. There are no NE Chondrichthyes in our dataset because the Spatial Data & Mapping Resources of the IUCN Red List dataset only contains evaluated species.

### 2.2 α‐ and β‐diversity

We estimated the number of species for each meter depth between 0 and 4000 m to obtain a vertical gradient of α‐diversity for all Actinopterygii and Chondrichthyes but also for each ocean (Figure S1). Next, we coupled this information with the IUCN Red List to assess the proportion of THR, LC, NE and DD species by depth. We partitioned the whole potential bathymetric range into five depth layers to study fish species composition changes according to depth: shallow (0–29 m), mesophotic (30–149 m), rariphotic (150–299 m; Baldwin et al., 2018), mesopelagic (300–999 m), and bathypelagic (1000–4000 m) depth layers (Figure S2). The bathymetric depth range was chosen to match with both coastal and pelagic environments.

We recorded the presence/absence of each species in each of these depth layers. To investigate compositional change, we used the two components of β‐diversity: (i) species turnover, which represents the replacement of species between depths and (ii) species nestedness, where depths with lower species richness would be subsets of those with higher species richness (Baselga, 2010). We calculated the turnover and nestedness components of β‐diversity by comparing the species composition of each pair of bathymetric ranges (e.g., Rocha et al., 2018). We used the Simpson pairwise dissimilarity index to calculate the turnover component and the nestedness‐fraction of the Sorensen pairwise dissimilarity with the R function beta.pair of the “Betapart” package (Baselga & Orme, 2012).

### 2.3 Species genetic sequence coverage

For the species genetic gap analysis, we considered 10,826 Actinopterygii species (61% of all marine Actinopterygii recorded in WoRMS) and 960 Chondrichthyes species (74% of all marine Chondrichthyes recorded in WoRMS) for which we have completed spatial and bathymetric information.

We downloaded the public genetic reference database ENA (Kanz et al., 2005) in June 2021 that we converted using *obiconvert* and extracted the sequences by in silico PCR using the *ecoPCR* from Obitools toolkit (Boyer et al., 2016) for the three markers: teleo (teleo forward primer—ACACCGCCCGTCACTCT, teleo reverse primer—CTTCCGGTACACTTACCATG; Valentini et al., 2016), MiFish‐U (MiFish‐U forward primer—GTCGGTAAAACTCGTGCCAGC, MiFish‐U reverse primer—CATAGTGGGGTATCTAATCCCAGTTTG) and MiFish‐E (MiFish‐E forward primer—GTTGGTAAATCTCGTGCCAGC, MiFish‐E reverse primer—CATAGTGGGGTATCTAATCCTAGTTTG; Miya et al., 2015) primer pairs. In silico PCRs are simulated PCR based on primer affinity for sequences. We allowed up to three mismatches on forward and reverse primers using *ecoPCR*. With this approach, we can only extract sequences which include the primer binding sites. Sequence entries which lack the primer binding sites cannot be extracted which might lead to an underestimation of species genetic coverage. To circumvent this issue, we complemented the *ecoPCR* output from the ENA database with a custom published database (Miya et al., 2020) for both MiFish‐U and ‐E markers since many sequences were available online and accessible via BLAST but could not be extracted using *ecoPCR* due to the lack of primer binding sites.

We then extracted the number of primer mismatches on each primer for each sequence obtained, except those from the custom MiFish database since primer sequences were not available. If there was some variability in the number of mismatches between individuals of the same species, we considered the one with the highest number of mismatches in a conservative approach. We compared all species amplified by the three primer pairs to fish checklists of the Atlantic, Indian, Pacific and Polar oceans, but also Mediterranean and Black seas and southeast Asia seas (Figure S1) to obtain the percentage of species sequenced by ocean or sea and depth (between 0 and 4000 m) for all primers. At each depth, we assessed the proportion of species with 0, 1, 2 or 3 mismatches on the forward and/or reverse primer teleo, MiFish‐U and MiFish‐E sequences amplified by in silico PCRs to estimate primer affinity, which influences the probability of sequence amplification.

Some species are “depth generalist”, i.e., they are recorded in the surface as well as in deeper layers. Other species, coined as “deep‐endemic” species in our study, are the one that have never been recorded in the shallow depth layer. To evaluate the genetic coverage of those “deep‐endemic” species, we classified each species according to their ability to ascend to a specific depth. This specific depth represents the minimal depth at which a given species can live.

## 3 RESULTS

### 3.1 Global bathymetric distribution of fish species

**FIGURE 1:**
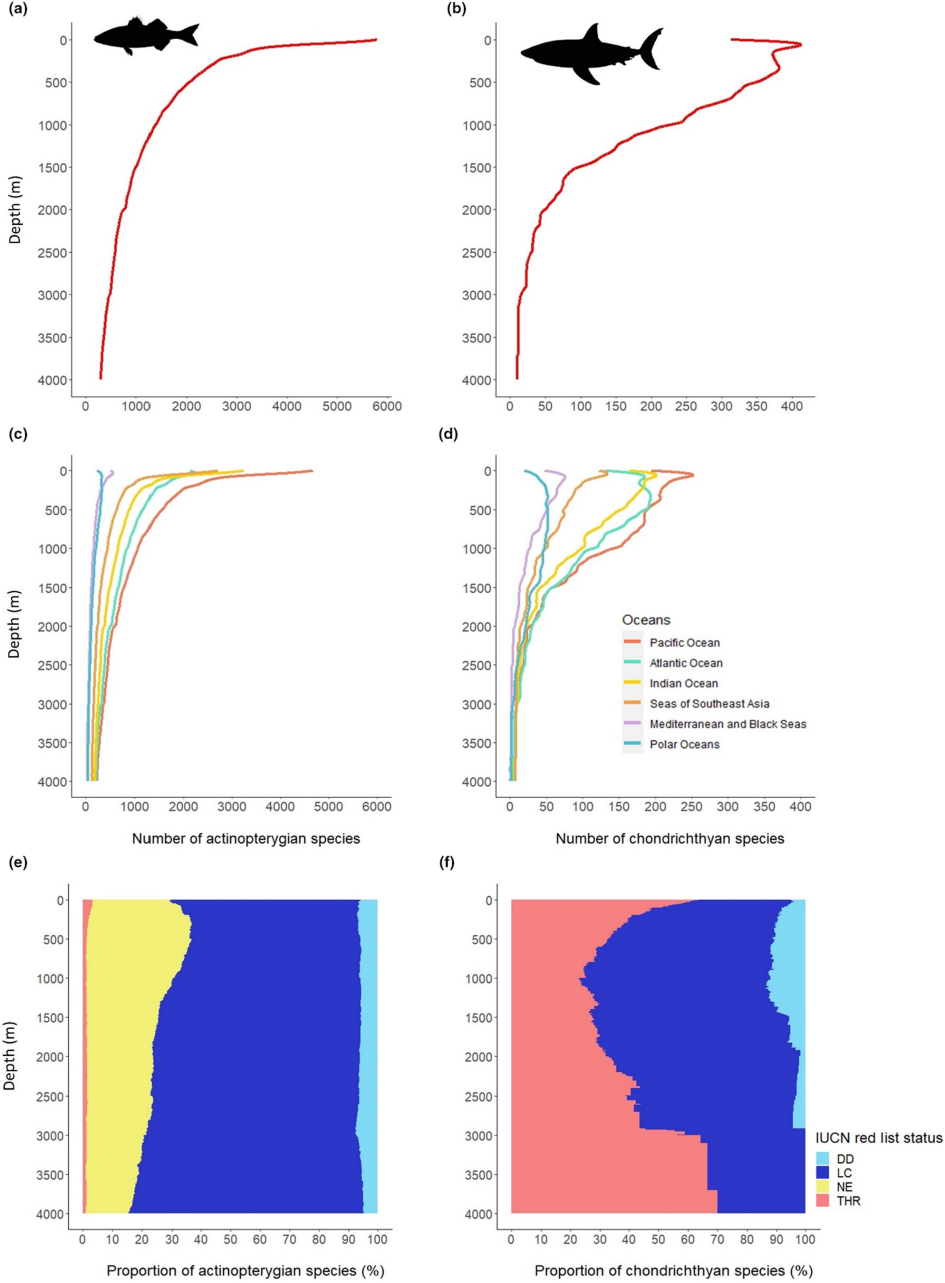
Number of (a) actinopterygian species by depth in all the oceans, (b) chondrichthyan species by depth in all the oceans, (c) actinopterygian species by depth and by ocean and (d) chondrichthyan species by depth and by ocean. (e) Proportion of THR, LC, DD and NE actinopterygian species by depth. (f) Proportion of THR, LC, NE and DD chondrichthyan species by depth

At the global scale, the number of Actinopterygii species is highest in the first 20 m where it reaches circa 6000 species and drops rapidly with increasing depth to reach around 300 species at 4000 m (Figure 1a). The number of Chondrichthyes also decreases with increasing depth but more slowly (Figure 1b). At 1000 m, four times fewer actinopterygian species are recorded compared with the first few meters. In comparison, only two times less chondrichthyan species are recorded at 1000 m compared with the first meters. This pattern is shared by the different oceans and seas considered in our study (Figure 1c,d). Yet, the decrease in fish species richness with depth is less marked for the polar oceans and the Mediterranean and Black seas. The difference in species richness between oceans is greater for shallow waters than for deep waters (Figure 1c,d).

The proportion of Actinopterygian species classified as THR from the IUCN red list is low at all depths (between 1% and 3% of species), but the proportion of DD species and NE species is high, with a maximum value of 40% at 250 m depth (Figure 1e). Contrary to Actinopterygii, the proportion of THR Chondrichthyes is very high while the proportion of DD species is low. The proportion of THR species decreases continuously between shallow (65%) and 1000 m depth (25%; Figure 1f). Below 2500 m the number of Chondrichthyes species is too low to make any interpretation.

**FIGURE 2:**
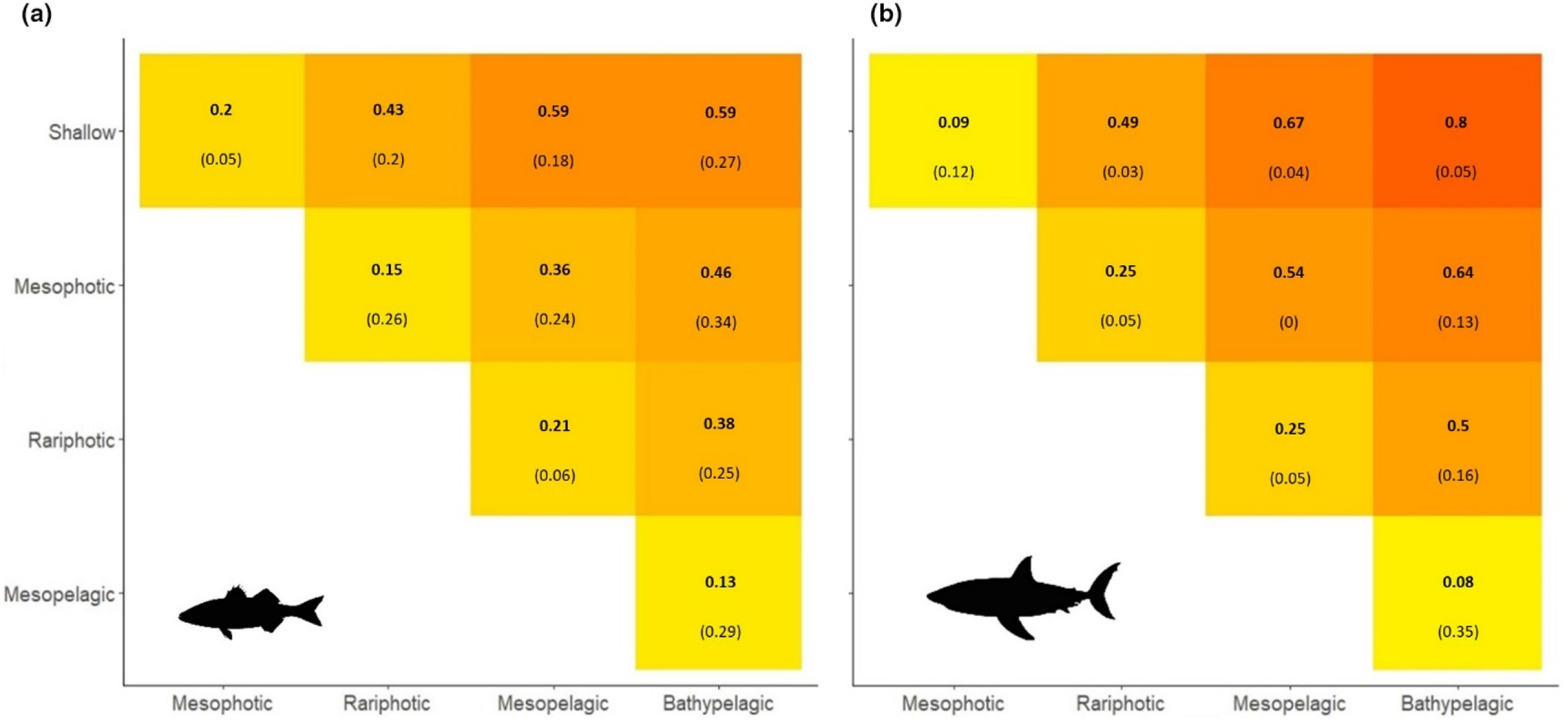
Species turnover (numbers in bold, Simpson pairwise dissimilarity) and nestedness components (numbers in brackets, nestedness‐fraction of the Sorensen pairwise dissimilarity) of β‐diversity for (a) actinopterygii and (b) chondrichthyes between depth layers. The diagonal contains the values of β‐diversity components between neighbor depth layers.

This expected drop in species richness with increasing depth, which explains the nestedness component of β‐diversity, is accompanied by a species turnover ranging between 0.13 and 0.59 for Actinopterygii and between 0.08 and 0.8 for Chondrichthyes (Figure 2). The highest turnover values between neighbor depth layers are observed between the rariphotic and mesopelagic layers for both Actinopterygii and Chondrichthyes but also between mesophotic and rariphotic layers for Chondrichthyes, suggesting that the rariphotic layer acts as a major boundary in the ocean. For Actinopterygii, shallow waters also host a unique composition since species turnover with other depth layers ranges between 0.2 and 0.59.

### 3.2 Bathymetric distribution of species genetic coverage

**FIGURE 3:**
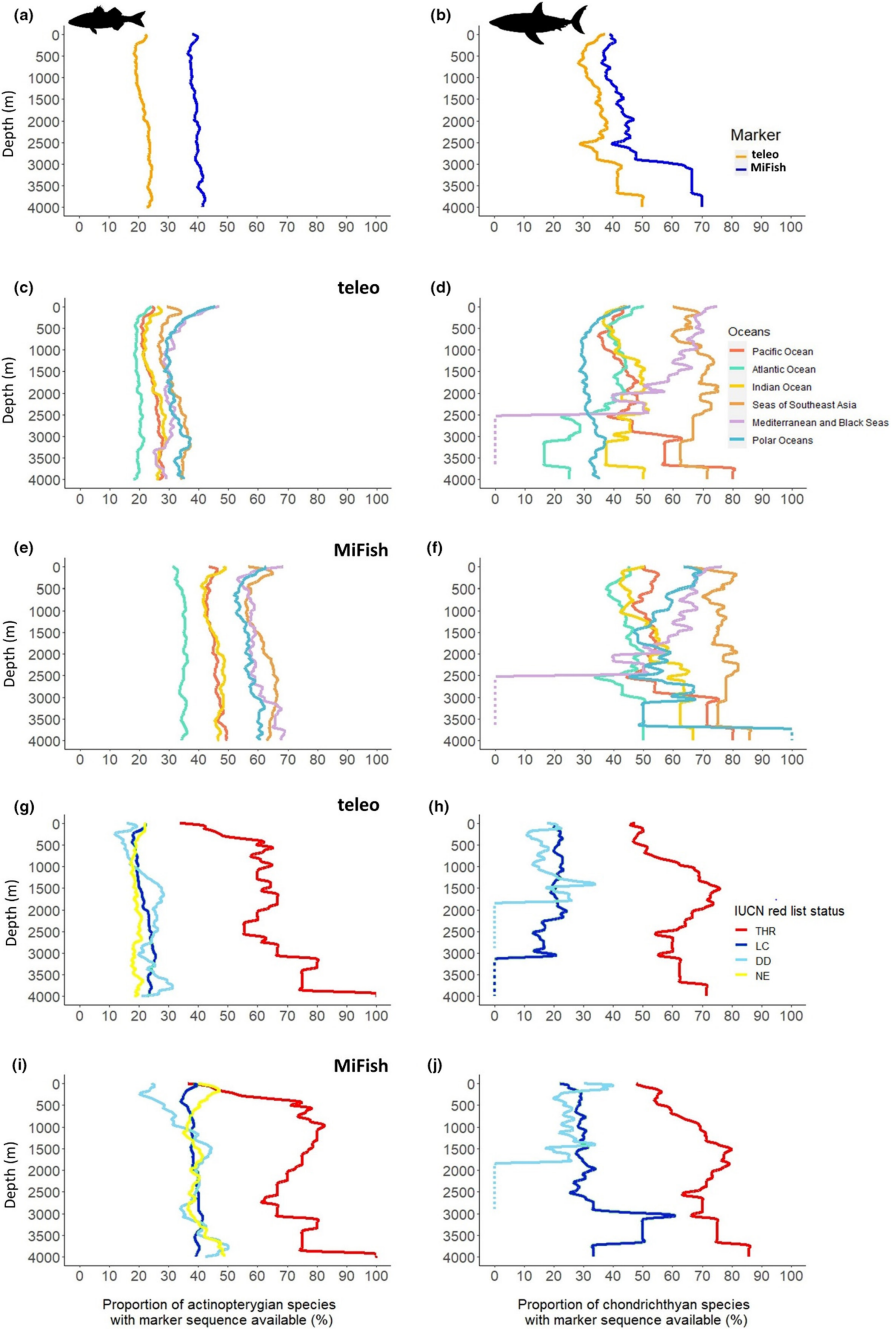
Genetic database coverage across all depth layers. Proportion of (a) actinopterygian and (b) chondrichthyan species with teleo or MiFish sequence available in the public genetic database. Proportion of (c) actinopterygian and (d) chondrichthyan species with teleo sequence available in the public genetic database per ocean. Proportion of (e) actinopterygian and (f) chondrichthyan species with MiFish sequence available in the public genetic database per ocean. Proportion of THR, LC, NE and DD (g) actinopterygian and (h) chondrichthyan species by depth with teleo sequence available in the public genetic databases. Proportion of THR, LC, NE and DD (i) actinopterygian and (j) chondrichthyan species by depth with MiFish sequence available in the public genetic databases

The diversity of marine fishes for which we have bathymetric and geographical distribution is not well covered in public genetic databases with 34% (3678 species) and only 18% (1963 species) of Actinopterygii covered by MiFish‐U/E and teleo markers, respectively. For Chondrichthyes, species coverage is 29% (282 species) and 25% (237 species), respectively, for the MiFish‐U/E and teleo markers. Two times more Actinopterygii species have a sequence available for MiFish compared with teleo in the public genetic database. Across depths, this difference in species genetic coverage remains similar: around 20% for teleo and 40% for MiFish (Figure 3a). For chondrichthyan species, species genetic coverage also remains similar across depths until 2500 m (Figure 3b). Across the oceans, species genetic coverage is weakly influenced by depth except for the teleo marker and Actinopterygii in the Mediterranean and Black Seas and polar oceans where species genetic coverage decreases from 45% to 30% in the first 500 m (Figure 3c–f). The Atlantic Ocean has the lowest species genetic coverage for Actinopterygii. At all depths, species genetic coverage is higher for THR species compared with DD, NE and LC species (Figure 3g–j). For both Actinopterygii and Chondrichthyes, the proportion of THR species with their sequence available increases with depth. For example, the proportion of THR Actinopterygii sequenced for the teleo marker increases from 35% in the first meters to 60% at 500 m depth (Figure 3g) and from 48% in the first meters to 75% at 1500 m depth for MiFish (Figure 3h).

**FIGURE 4:**
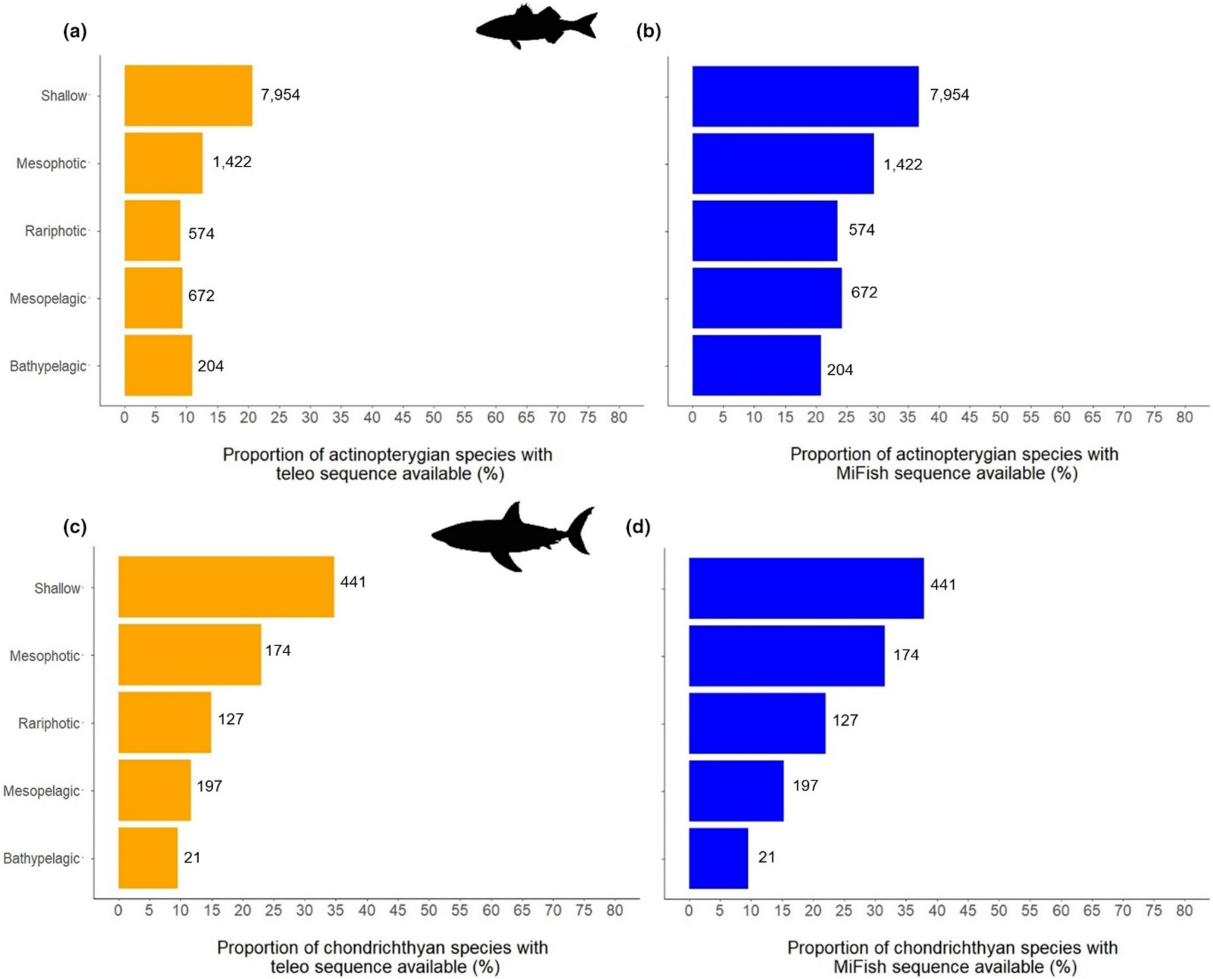
Number of actinopterygian species that can ascend to the shallow, mesophotic, rariphotic, mesopelagic or bathypelagic depth layer (number of species on right of the bars) and their coverage (*x*‐axis) in the genetic reference database for the teleo (a) and MiFish (b) markers. Number of chondrichthyan species that can ascend to the shallow, mesophotic, rariphotic, mesopelagic or bathypelagic depth layer (number of species on right of the bars) and their coverage (*x*‐axis) in the genetic reference database for the teleo (c) and MiFish (d) markers.

Among Actinopterygii, 7954 species can ascend to the shallow depth range, 1422 species to the mesophotic range, 574 species to the rariphotic range, 672 species to the mesopelagic range and 204 species to the bathypelagic range. Among Chondrichthyes, 441 species can ascend to the shallow depth range, 174 species to the mesophotic range, 127 species to the rariphotic range, 197 species to the mesopelagic range and 21 species to the bathypelagic range. The “deep‐endemic” species (species not recorded in the shallow depth layer but only in mesophotic, rariphotic, mesopelagic or bathypelagic layers) are less covered than those that can ascend to the shallow depth range (0–30 m; Figure 4). For Actinopterygii, less than 10% of the 574 species occurring only from rariphotic to deeper layers have a teleo marker sequence available while more than 20% of the 7954 species that can ascend to the shallow depth range have a teleo sequence available (Figure 4a).

**FIGURE 5:**
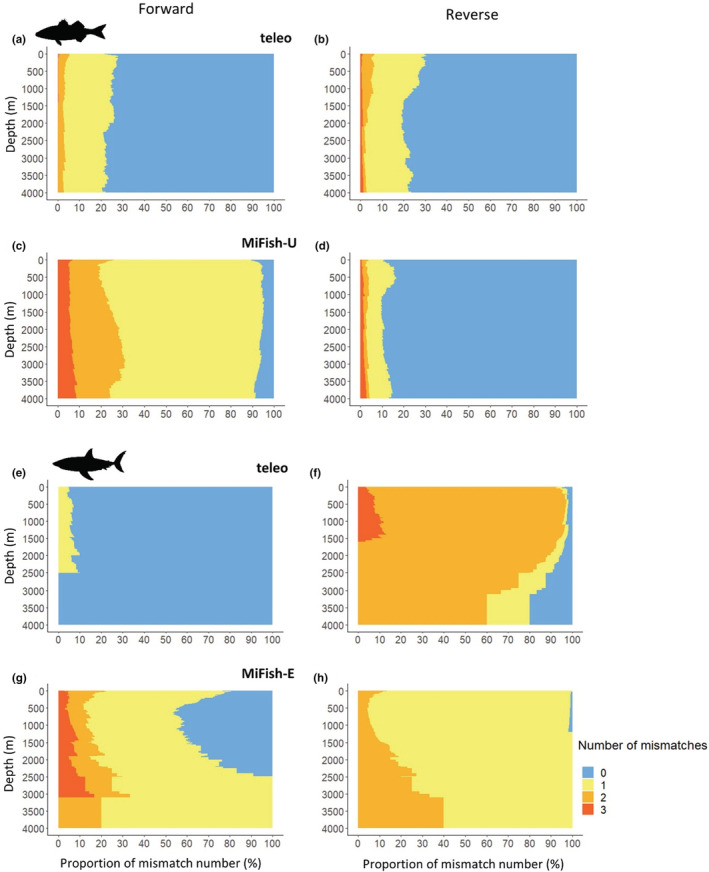
Number of primer mismatches for (a) teleo forward primer, (b) teleo reverse primer, (c) MiFish‐U forward primer and (d) MiFish‐U reverse primer for Actinopterygii. Number of primer mismatches for (e) teleo forward primer, (f) teleo reverse primer, (g) MiFish‐E forward primer and (h) MiFish‐E reverse primer for Chondrichthyes

For Actinopterygii, the number of mismatches on forward and reverse teleo primers is for almost all species sequenced with around 80% species at all depths having 0 mismatch (Figure 5a,b). For MiFish‐U, there are few mismatches on the reverse primer for most species with around 90% having 0 mismatch at all depths but there are more mismatches on the forward primer with around 70% of species having one mismatch and 20% having two or three mismatches at all depths (Figure 5c,d). Actinopterygii species found at low depth do not display more primer mismatches than their shallower counterparts for any marker.

For Chondrichthyes, almost all sequenced species have 0 mismatch on the forward teleo primer while they have 0, 1, 2 or 3 mismatches on the reverse teleo primer (Figure 5e,f). The number of mismatches on the reverse MiFish‐E primer is 1 or 2 for almost all sequenced species (Figure 5h) while it ranges between 0 and 3 for most species with only 0 and 1 mismatch for the forward MiFish‐E primer. Deep chondrichthyan species do not have more primer mismatches than shallow species for all markers.

## 4 DISCUSSION

Our species depth range analysis shows a strong decrease in species richness with increasing depth. This is consistent with the global pattern observed by Costello and Chaudhary (2017) on all marine taxa combined. At smaller geographical and bathymetric scale, this fish species richness pattern has also been observed around the Bermuda archipelago (Stefanoudis, Gress, et al., 2019) and in the Tsitsikamma National Park Marine Protected Area (South Africa; Heyns‐Veale et al., 2016). The strong decrease in actinopterygian and chondrichthyan species richness with increasing depth (Figure 1a,b) can be explained by several phenomena. Firstly, fish species' bathymetric fundamental niche is constrained by their physiological ability to support abiotic conditions of the deep‐sea: low temperature, high pressure, scarce food, darkness and low oxygen availability (Spence & Tingley, 2020; Treberg & Speers‐Roesch, 2016). Few species might be adapted to these harsh conditions. Secondly, the deep ocean has been underexplored and is under‐represented in global databases of marine biological records (Webb et al., 2010). Many deep‐sea species have certainly not yet been discovered (Costello et al., 2012), and the depth range of some known species may have been underestimated. Moreover, these fish biodiversity patterns are not homogenous among the oceans (Figure 1c,d). Difference in species richness between oceans is much higher at shallow depth than in deep waters. The decrease in fish richness with increasing depth is steeper in the Pacific Ocean, Indian Ocean and seas of southern Asia than in the Polar oceans and in the Mediterranean and Black Seas. It can be partly explained by the development of a complex mosaic of coral reef habitats in shallow waters of tropical regions which has been a major driver of cladogenesis (Cowman & Bellwood, 2011). For example, reef‐associated Scaridae (89 species in our data set) and Chaetodontidae (122 species in our data set) families have, respectively, 94% and 77% of their species exclusively living in the first 100 m. In addition to the high diversification rate, large‐scale spatial patterns of species richness can be explained by the difference in time‐for‐speciation, related to geological and colonization history (Miller et al., 2018). The present‐day actinopterygian species richness bathymetric gradient is the result of ancient diversification and colonization events within deep‐sea and shallow habitats over the past 200 million years (Miller et al., 2022).

Conversely to Actinopterygii, Chondrichthyes species richness is still high in the mesophotic and in the rariphotic zones until 400 m depth (Figure 1b,d) but communities change gradually (Figure 2b). As already described (Musick & Cotton, 2015; Priede et al., 2006; Treberg & Speers‐Roesch, 2016), we show that this taxonomic group is almost absent in abyssal regions below 3000 m and uncommon below 2000 m. To explain this observation, Priede et al. (2006) suggest that even if they are well‐adapted to life at high pressure, Chondrichthyes are excluded from the abyss because of their high‐energy demand which cannot be sustained in abyssal conditions. The proportion of threatened and near threatened chondrichthyan species decreases with increasing depth, suggesting that either deep‐sea species have been up to now less impacted by human activities or are just currently unknown to be impacted as the deep‐sea remains difficult to monitor and time series are lacking. A quarter of all chondrichthyan species is nevertheless threatened or near threatened at 1000 m depth while deep‐sea Chondrichthyes are considered as highly vulnerable to fishing since having slow population recovery rates (Simpfendorfer & Kyne, 2009). Deep‐sea fishes generally grow slowly, live longer and have later age at maturity and a lower fecundity (Drazen & Haedrich, 2012). These life history traits make them more vulnerable to human disturbances (Mindel et al., 2018). This highlights the importance of protecting shallow waters but also mesophotic and deep‐sea layers which have unique fish communities (Levin et al., 2017).

Beyond the level of α‐diversity, the amount of species turnover between adjacent depth layers, or β‐diversity, is key to the resilience of marine ecosystems owing to connectivity where refugia can act as sources of individuals toward impacted layers (Bongaerts et al., 2010). For instance, it has been shown that mesophotic coral ecosystems can act as a refugia for some exploited species (Lindfield et al., 2015). Chaikin et al. (2022) describe a deepening shift pattern for Chondrichthyes but a depth range shrinkage when sea surface temperature increases in the Mediterranean Sea, suggesting a capacity of deep layers to act as refugia under ocean warming. However, the rapid diminution of Actinopterygian species richness with increasing depth (Figure 1a,c) coupled to a gradual species turnover across depths (Figure 2a) suggests a limited ability of mesophotic layers to act as refugia for many species. Rocha et al. (2018) also refuted the mesophotic refugia hypothesis by revealing, from in situ observations, distinct fish communities between the mesophotic and shallow marine ecosystems near the Bahamas. Specifically, the species turnover component of β‐diversity calculated with the Jaccard's dissimilarity index between adjacent depth ranges is comprised between 0.5 and 0.75. Yet, this unexpected high species turnover may be partly explained by the lack of data and the underestimation of α‐diversity in the mesophotic reefs, particularly for rare, elusive and cryptic species (Muff et al., 2022). For example, Brandl et al. (2018) report that half of cryptic species are ignored in classical surveys. So, species turnover may be underestimated in our analysis because of uncertainty in our species depth ranges which are based on records with potential outliers. So even if we removed the 1% higher and lower recorded depths, we may still overestimate depth range so underestimate β‐diversity. Some species depth ranges extracted from Fishbase might also be overestimated. Thus, new sampling methods, able to assess more exhaustively fish marine biodiversity and to operate in the deep sea, are urgently needed to better address the question of whether mesophotic ecosystems can act as a refugia for shallow marine waters under threats. Yet, our knowledge in geographic, depth range and conservation status of fish species, which are of primary importance for marine conservation, is still very incomplete, imprecise, and biased toward some species groups (Menegotto & Rangel, 2018; Miqueleiz et al., 2020).

eDNA has been shown to fill biodiversity inventory gaps for still poorly known habitats such as mesophotic ecosystems (Muff et al., 2022), and species groups like cryptobenthic fishes (Boulanger et al., 2021; Mathon et al., 2022). eDNA metabarcoding is thus a promising tool to detect fish species occurrences in the deep‐sea (Canals et al., 2021). One of the principal limitations in the use of eDNA metabarcoding for inventories is the incompleteness of genetic reference databases. Global species genetic coverage is low for fish species (Marques et al., 2021) but is weakly influenced by depth for the teleo and MiFish‐U/E markers (Figure 3c–f). However, by distinguishing “deep‐endemic” species from “depth generalist” species we show that “deep‐endemic” species are less covered than those that can ascend to the shallow depth range (0–30 m; Figure 4) while eDNA could provide a relevant tool for their monitoring. We thus need to focus our efforts on sequencing “deep‐endemic” fish species. Our analysis also reveals that THR species have a better genetic coverage than LC, DD and NE species. The number of threatened species estimated by the IUCN Red List is likely underestimated. So, a lot of DD and NE species might be threatened (Dulvy et al., 2021). We argue that an important sequencing effort should be dedicated to DD species and not yet evaluated species to improve their monitoring through eDNA metabarcoding.

Beyond the incompleteness of genetic reference databases, some species can be missed when applying eDNA metabarcoding due to mismatches between primers and the target sequences (Elbrecht et al., 2018). Our analysis on the number of primer mismatches for MiFish‐U/E and teleo metabarcoding primers demonstrates that these primers are equally well‐adapted for both shallow water and deep‐sea fish species detection (Figure 5). So, in theory, teleo and MiFishU/E are valid markers to study deep‐water fish communities with eDNA metabarcoding. Our study only considers in silico PCR results to assess global species genetic coverage. However, some species theoretically detected by in silico PCR may remain undetected by real PCR even if they occur in the sampling site. For example, four‐armed frogfish (*Tetrabrachium ocellatum*) is unlikely to be amplified by real PCR with the teleo primer pair due to its high number of mismatches (three mismatches on forward and three mismatches on reverse primer). For this species, another primer pair such as Vert01 12S should be used (Riaz et al., 2011). Stat et al. (2017) highlight the need to use multi‐marker approaches to better evaluate biodiversity through eDNA metabarcoding. Moreover, our approach did not include the mismatch position, which is important in the probability of species sequence amplification beyond the number of mismatches. Mismatches at the last two positions of the 3′ end of a primer are known to be particularly detrimental to PCR amplification (Zhang et al., 2020), so studies focusing on deep‐water species should pay a particular attention to the location of potential mismatches for the primer pair chosen to identify any amplification bias toward their group of interest. Another limitation for species assignment using eDNA metabarcoding is the marker taxonomic resolution. Some closely related species share the same marker sequence like *Dentex gibbosus*, *Pagrus auriga* and *Dentex dentex* (Sparidae) which have all the same teleo 12S sequence, preventing assignment at the species level. Another limitation in the use of eDNA to study fish depth distribution is the potential eDNA passive vertical transport which remains understudied. There is some evidence suggesting that this phenomenon is limited (Jeunen et al., 2020; Monuki et al., 2021). However, Canals et al. (2021) have highlighted a vertical transport of eDNA from upper to deeper layers through the sinking process of organic matter (scales, feces, or corpses among others).

The future improvement in reference databases requires collaborating with aquariums and museums to sequence some fish species like deep‐sea fishes or rare species which are difficult to capture in the wild. Extraction of ancient DNA from museum samples is challenging but the advances in this field are promising (Silva et al., 2019) and initiatives such the one of Margaryan et al. (2020) for vertebrates in Denmark open new perspectives for the development of genetic databases. Currently, few studies (Gaither et al., 2018; Tenggardjaja et al., 2014) have focused on the vertical connectivity of fish species and their potential ecotypes across depth which is a key information in conservation. Collecting genetic samples of fish individuals at different depths is often challenging and invasive. The emergent field of eDNA‐based population genetics could have a great potential to study fish population connectivity across depths (Adams et al., 2019). For instance, eDNA has been recently used to investigate diversity within populations of Striped red mullet (*Mullus surmuletus*; e.g., Macé et al., 2022), or Blackfoot pāua (*Haliotis iris*; Adams et al., 2022), and to successfully estimate population genetic differentiation like for the whale shark (Sigsgaard et al., 2016). Only through standard, sustained and multi‐objectives observations at all depths we could get the insights needed to understand the fundamental ecological processes that govern the dynamics of marine ecosystems, and design depth‐specific strategies to effectively protect them in the future. eDNA has the potential to fulfill these requirements by providing estimates of α‐ and β‐diversity for species, taxa, MOTUs and genes.

## AUTHOR CONTRIBUTIONS

**Agnès Duhamet:** Conceptualization (equal); data curation (lead); formal analysis (equal); investigation (lead); methodology (lead); resources (equal); software (equal); writing – original draft (lead); writing – review and editing (equal). **Camille Albouy:** Formal analysis (supporting); methodology (supporting); supervision (supporting); writing – review and editing (equal). **Virginie Marques:** Methodology (supporting); resources (supporting); software (supporting); writing – review and editing (equal). **Stephanie Manel:** Supervision (supporting); writing – review and editing (equal). **David Mouillot:** Conceptualization (equal); investigation (supporting); supervision (lead); writing – review and editing (equal).

## CONFLICT OF INTEREST

All authors declare that there is no conflict of interest regarding the publication of this article.

## Supporting information


FigureS1‐S2

## Data Availability

Data are available on Figshare. https://doi.org/10.6084/m9.figshare.20403111.
