# Supplementary material for: The global depth range of marine fishes and their genetic coverage for environmental DNA metabarcoding
Source: Ecol Evol. 2023 Jan 18;13(1):e9672. doi: 10.1002/ece3.9672 (PMC9846838; doi:10.1002/ece3.9672)
Supplement: Supplementary file 1 — FigureS1‐S2 [file ECE3-13-e9672-s001.docx]

**SUPPORTING INFORMATION**


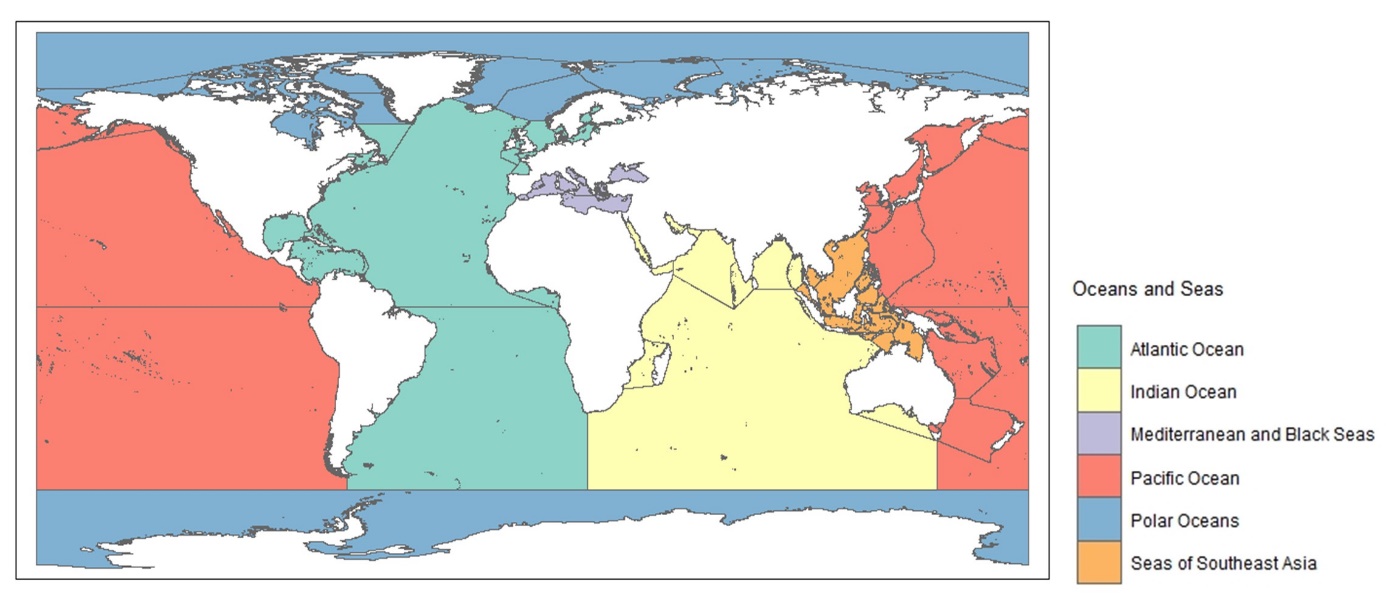


**Figure S1:** Seas and oceans delineation


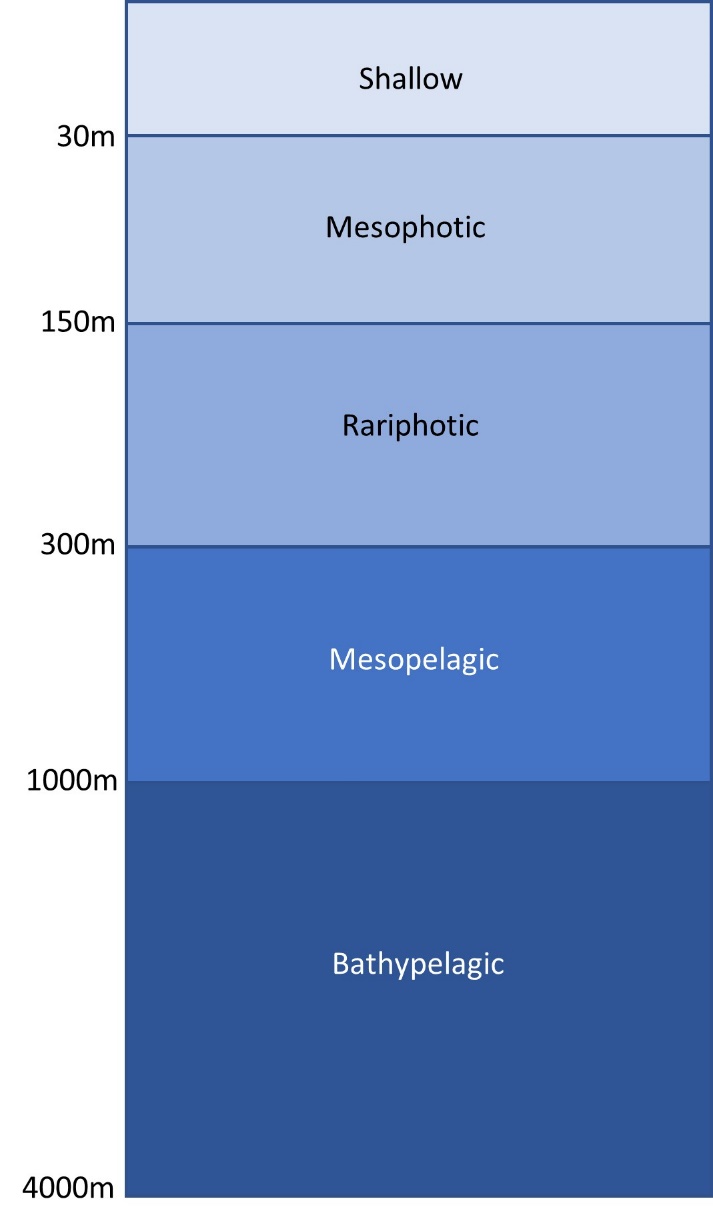


**Figure S2:** Delineation of the five depth layers
